# Relationship between molecular markers and important fruit‐related traits in almond (*Prunus dulcis* [Mill.] D.A. Webb syn. *P. amygdalus* Batsch) as revealed using multiple regression analysis (MRA)

**DOI:** 10.1002/fsn3.3656

**Published:** 2023-09-01

**Authors:** Ali Khadivi, Zeinab Mashhadi, Akram‐Sadat Hosseini

**Affiliations:** ^1^ Department of Horticultural Sciences, Faculty of Agriculture and Natural Resources Arak University Arak Iran

**Keywords:** almond, breeding, fruit characteristics, molecular markers, regression analysis

## Abstract

Almond (*Prunus dulcis* [Mill.] D.A. Webb syn. *P. amygdalus* Batsch) is one of the most important nut crops, and its kernel is the edible part that has a high nutritional value and is used in the confectionery and cosmetics industries. The present research aimed to identify random amplified polymorphic DNA (RAPD) and inter simple sequence repeat (ISSR) molecular markers associated with important fruit traits in late‐blooming almond genotypes through multiple regression analysis (MRA). The studied genotypes showed significant differences from each other in terms of the measured fruit‐related traits. The ISSR primers used produced a total of 125 bands in the studied germplasm, of which 112 showed polymorphic bands. The RAPD primers produced a total of 190 DNA fragments, of which 172 fragments showed polymorphism among genotypes. Some polymorphic fragments of ISSR and RAPD showed significant correlations with the fruit traits measured. Some of these informative markers were associated with more than one trait, which could be caused by the pleiotropic effects of quantitative trait loci related to each other in different traits. For instance, some of the markers showed significant correlations with both nut weight and kernel weight, which indicates a positive correlation between these two traits. Informative markers identified in this study can be used to select suitable parents for population generation for mapping. It is also useful for selecting superior genotypes, especially when information about their genetic basis, such as a linkage map, is not available.

## Graphical abstract

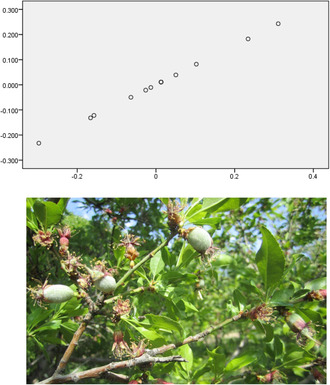

## 1 INTRODUCTION

Almond (*Prunus dulcis* [Mill.] D.A. Webb syn. *P. amygdalus* Batsch) is native to Central and Western Asia. Iran has a considerable variety of almond germplasm with cultivated almonds that have grown in different climatic conditions of the country. Kernel is the edible part of this nut crop that has a high nutritional value and is used in the confectionery and cosmetics industries. Almonds are not only an important source of macronutrients such as lipids, proteins, fiber, and minerals but also a very important source of specific plant substances such as vitamin E (α‐tocopherol), folate, and oleic acid (Kodad & Rafel Socias i Company, 2008). As many desirable almond cultivars have been introduced through the selection of superior native genotypes, the native germplasm is certainly considered the basis for the improvement and introduction of fruit tree cultivars (Mehlenbacher, 2003).

In the past few years, molecular markers based on DNA have been widely used for various purposes in plants (Langridge et al., 2001). Constant progress in improving plant species breeding depends on the genetic diversity of plants. Therefore, identification and management of this diversity are necessary for reform programs. In addition, knowledge of genetic diversity makes it easier to manage the protection of plant germplasm.

Although mapping based on quantitative trait loci (QTL) is suitable for tracking genes related to these traits, this process is time‐consuming and laborious (Rakshit et al., 2010). To overcome these limitations, it seems appropriate to identify markers dependent on traits through regression. Multiple regression analysis (MRA) based on the relationship between molecular markers, as independent variables, and morphological traits, as dependent variables, is a suitable method to identify trait‐dependent markers. This analysis determines the determination coefficient (*r* ^2^), which shows the relationship between the morphological trait and the molecular marker (Gomez & Gomez, 1984).

The availability of a large number of molecular markers and morphological traits can help study the regression analysis between these markers and morphological traits. Most of the relational analysis studies based on continuity have made it possible to track different markers related to morphological traits, but often due to the large distance between the marker and the morphological trait, selection is made with the help of the associated marker, as well as isolation and assimilation. It has made finding the desired gene difficult, and in addition, only a small number of genotypes have been used as parents for gene mapping. To overcome this problem, regression analysis between markers and morphological traits has been used, which not only enables the mapping of genes with a higher degree of confidence but also the identification of markers that are on the map. It enables detection based on untraceable linkage (Langridge et al., 2001; Neale & Savolainen, 2004; Roy et al., 2006).

Molecular markers have been used to determine trait‐related markers in some plants. For instance, the relationship between the data obtained from different molecular techniques and biochemical traits has been investigated in *Morus* spp. (Kar et al., 2008) and *Valeriana jatamansi* (Jugran et al., 2013). Also, the relationship of molecular markers with morphological traits has been investigated in cotton (Shen et al., 2007; Zeng et al., 2009), wheat (Ma et al., 2005), maize (Song et al., 2004), *Morus laevigata* (Chatterjee et al., 2004), pomegranate (Basaki et al., 2011), and sweet cherry (Khadivi‐Khub, 2014).

The RAPD (random amplified polymorphic DNA) technique is based on the replication of random DNA fragments and does not require knowledge of the DNA sequence of the template. Other advantages of this technique include low cost, simplicity, high speed, and the need for a small amount of DNA (Chatterjee et al., 2004). In addition, the ISSR (inter simple sequence repeat) technique includes the use of microsatellite sequence as a primer in polymerase chain reaction to amplify polyallelic markers. This technique is simple, fast, and useful (Khadivi‐Khub, 2014).

A wide diversity of almond germplasm in terms of quantitative and qualitative characteristics has been reported (Khadivi, Goodarzi, & Sarkhosh, 2019; Khadivi, Safdari, et al., 2019; Khadivi‐Khub & Etemadi‐Khah, 2015). Iran also has a high variation of almonds, but the regression relationship of morphological traits with molecular markers has not been investigated for this species. Therefore, the present research aimed to identify RAPD and ISSR markers associated with important fruit morphological traits in 76 late‐blooming almond genotypes using MRA. The result of this study helps identify the genes responsible for the emergence of important traits that can be used in breeding programs.

## 2 MATERIALS AND METHODS

### 2.1 Plant material

In this study, 76 late‐blooming almond genotypes were selected from Hezaveh and Khondab areas in the Arak region, Markazi province, Iran. The genotypes examined were named according to their location and the names were supplemented with the numerical characters. The selected genotypes were mature (10–12 years old) and healthy, and had a full crop. Common orchard management, including irrigation, nutrition, and pest and disease control, was regularly done.

### 2.2 Fruit‐related trait evaluation

Fruits were harvested randomly from different parts of the trees. The evaluated traits included the traits related to the nut (length, width, and weight) and the traits related to the kernel (length, width, weight, and percentage). Traits were evaluated on 50 nuts of each genotype over two years. The traits related to nut and kernel dimensions were measured using a digital caliper. Also, the traits related to nut and kernel weight were measured using an electronic scale (Lutron, GM‐500) with a precision of 0.01 g.

### 2.3 Molecular evaluation

DNA extraction from mature and fresh leaf samples was done using the method of Doyle and Doyle (1987). The quantity and quality of the obtained DNA were determined using spectrophotometry at 260 and 280 nm wavelengths and DNA electrophoresis in agarose gel with a concentration of 1.00%, and with their help, the same concentration of DNA (5 ng μL^−1^) was determined. Sterile, double‐distilled water was used for dilution. The PCR reaction for the RAPD technique was based on the method described by Khadivi‐Khub et al. (2008), and the reaction for the ISSR technique was based on the method used by Thimmappaiah et al. (2009). For each reaction mixture, 1 μL of DNA prepared with a concentration of 5 ng μL^−1^ was added to 24 μL of the PCR reaction mixture, including dNTPS, PCR buffer, *Taq* DNA polymerase, primer, and MgCl_2_ (Cinagen Company, Iran), and sterile double‐distilled water was added. Finally, the volume of the PCR reaction solution reached 25 μL and the resulting mixture was subjected to the polymerase chain reaction in a Bio‐Rad thermal cycler model I‐Cycler.

For the ISSR technique, the polymerase chain reaction was started with the initial annealing of genomic DNA at 94°C for four minutes and 35 cycles including 92°C for one minute for annealing. Binding of primers to the template strand for 50 s (the temperature of primer binding varied from 48 to 57°C depending on the primer), extension of the new strand for one minute at 72°C, and final extension at the temperature of 72°C were followed for 10 min.

For the RAPD technique, thermal cycles include 94°C for four minutes for an initial annealing, 35 cycles at 92°C for one minute for annealing, an annealing temperature of 37°C for one minute, the temperature of the reproduction of the fragments at 72°C, for two minutes, and finally a temperature cycle of 72°C for five minutes to complete the expansion. PCR product electrophoresis was analyzed in agarose gel with a concentration of 1.50%.

### 2.4 Statistical analysis

For fruit traits, means were compared using SAS software and Duncan's test. For molecular analysis, after performing the ISSR and RAPD tests to check the genetic polymorphism between genotypes, the presence of a specific band was given as 1, and its absence was given 0. The size of the amplified bands was estimated using Quantity One software. For each primer, the total number of produced bands, the number of polymorphic bands, and the percentage of polymorphism were calculated.

Stepwise regression analysis was done to trace the relationship between the tested morphological traits as dependent variables and RAPD and ISSR markers as independent variables using SPSS ver. 16. The *r* ^2^ and *β* coefficients were calculated using regression analysis and investigated for different markers related to traits. The *r* ^2^ is the multiple‐justified correlation coefficient that is calculated for each marker and indicates the correlation of the marker with the morphological trait. Also, *β* is the standardized regression coefficient, which is calculated by MRA for each trait‐related marker (Kar et al., 2008).

## 3 RESULTS

### 3.1 Fruit‐related trait analysis

The studied genotypes showed significant differences from each other in terms of the measured fruit‐related traits (Table 1). Traits with a high coefficient of variation have a wider range of trait quantity, which is considered a wider range of selection for that trait. Nut length varied from 20.53 to 42.70 mm, and nut width ranged from 15.42 to 26.49 mm. Nut weight ranged from 1.28 to 6.68 g, with an average of 3.31. Kernel length ranged from 12.74 to 27.14 mm, and kernel width varied from 7.97 to 18.52 mm. Kernel weight ranged from 0.39 to 1.46 g, with an average of 0.88 g. Kernel percentage varied from 10.42% to 47.89%.

**TABLE 1:** The values of the most important fruit traits for the studied late‐blooming almond genotypes.

Genotype	Nut length (mm)	Nut width (mm)	Nut weight (g)	Kernel length (mm)	Kernel width (mm)	Kernel weight (g)	Kernel percentage (%)
Hezaveh‐1	20.53	16.40	1.52	15.49	10.74	0.64	41.67
Hezaveh‐2	31.93	23.18	5.22	22.63	14.97	1.01	19.38
Hezaveh‐3	28.65	19.85	3.88	21.63	12.54	0.97	24.93
Hezaveh‐4	27.89	16.38	2.12	18.73	9.53	0.63	29.84
Hezaveh‐5	29.09	17.02	2.22	21.10	10.96	0.64	28.64
Hezaveh‐6	25.34	18.03	2.12	19.16	11.64	1.02	47.89
Hezaveh‐7	26.58	16.08	2.11	18.30	11.43	0.64	30.30
Hezaveh‐8	29.92	16.68	2.36	20.38	11.58	1.01	42.80
Hezaveh‐9	30.67	19.07	3.29	22.76	12.25	0.91	27.69
Hezaveh‐10	30.85	23.09	4.47	18.18	11.58	1.31	29.30
Hezaveh‐11	31.70	21.04	3.60	22.32	12.93	0.96	26.66
Hezaveh‐12	28.61	19.13	3.28	20.83	12.12	0.83	25.37
Hezaveh‐13	26.87	17.29	2.38	18.41	11.17	0.62	26.26
Hezaveh‐14	39.17	15.42	1.55	12.74	7.97	0.39	24.94
Hezaveh‐15	24.48	15.43	1.65	16.42	10.55	0.55	33.56
Hezaveh‐16	22.66	17.21	1.87	16.33	10.11	0.67	35.82
Hezaveh‐17	29.13	22.09	4.16	20.62	13.83	1.00	23.99
Hezaveh‐18	31.54	20.90	3.67	20.04	10.89	1.12	30.41
Hezaveh‐19	28.67	20.18	2.69	21.32	12.96	0.52	19.48
Hezaveh‐20	27.21	17.75	2.21	21.53	11.58	0.78	35.48
Hezaveh‐21	21.16	15.69	1.28	15.42	9.19	0.43	33.46
Hezaveh‐22	28.69	18.97	2.54	21.37	12.23	0.85	33.46
Hezaveh‐23	31.62	23.54	4.11	23.46	14.42	1.19	29.06
Hezaveh‐24	33.74	21.08	4.04	23.48	13.06	0.75	18.65
Hezaveh‐25	28.01	17.44	2.26	22.42	10.71	0.70	30.96
Hezaveh‐26	28.14	20.32	3.52	19.59	12.74	1.03	29.29
Hezaveh‐27	28.01	19.39	3.44	21.02	11.99	0.63	18.20
Hezaveh‐28	30.54	19.50	3.50	22.25	11.82	0.78	22.21
Hezaveh‐29	24.94	17.54	2.21	19.06	10.08	0.67	30.40
Hezaveh‐30	32.15	21.60	4.17	23.71	12.93	1.13	27.00
Hezaveh‐31	30.11	18.45	3.05	23.15	10.85	0.79	26.00
Hezaveh‐32	27.19	16.37	2.84	19.54	10.40	1.12	39.44
Hezaveh‐33	27.90	16.62	3.29	22.83	10.34	0.82	25.02
Khondab‐1	28.89	16.43	2.28	22.49	11.55	0.72	31.36
Khondab‐2	28.94	18.16	3.20	21.90	9.77	1.40	43.76
Khondab‐3	28.19	20.44	2.80	19.35	11.08	1.19	42.64
Khondab‐4	28.09	20.66	3.26	20.80	11.38	1.00	30.78
Khondab‐5	29.14	20.85	6.68	19.23	11.44	0.92	13.77
Khondab‐6	29.80	21.86	4.78	20.99	13.15	1.46	30.58
Khondab‐7	26.63	18.62	2.49	19.67	11.10	0.94	37.82
Khondab‐8	26.81	19.20	2.84	20.44	10.62	0.82	28.82
Khondab‐9	25.52	17.01	2.49	17.03	11.42	0.63	25.24
Khondab‐10	30.68	18.99	4.15	22.31	10.86	0.82	19.67
Khondab‐11	25.46	18.65	2.13	20.76	12.21	0.78	36.74
Khondab‐12	28.56	21.98	3.79	22.40	10.66	0.98	25.87
Khondab‐13	28.06	19.20	3.21	22.15	12.25	0.99	30.76
Khondab‐14	29.28	22.73	4.62	22.57	12.57	1.21	26.22
Khondab‐15	32.75	21.59	4.58	23.17	12.55	1.11	24.23
Khondab‐16	29.19	17.85	3.05	21.45	10.40	0.92	30.18
Khondab‐17	26.15	19.06	2.84	20.70	18.52	0.91	32.14
Khondab‐18	28.52	20.18	3.42	20.76	10.64	0.90	26.36
Khondab‐19	29.83	20.05	4.35	23.42	13.06	1.00	23.02
Khondab‐20	30.41	18.67	2.99	23.55	14.29	0.89	29.81
Khondab‐21	37.36	20.53	4.45	26.82	12.84	0.98	21.95
Khondab‐22	32.21	23.04	5.53	23.89	12.47	1.19	21.57
Khondab‐23	33.14	22.77	5.12	22.47	11.98	1.18	23.07
Khondab‐24	27.30	18.73	2.94	18.11	11.58	0.63	21.50
Khondab‐25	30.11	20.51	2.41	17.62	10.26	0.51	21.03
Khondab‐26	31.63	17.55	3.01	21.72	10.91	0.91	30.16
Khondab‐27	29.40	17.50	3.03	20.23	10.75	0.65	21.41
Khondab‐28	30.94	21.76	4.36	16.16	10.47	0.45	10.42
Khondab‐29	27.11	24.37	5.41	17.46	14.34	1.44	26.65
Khondab‐30	34.37	26.49	5.66	21.57	14.19	0.96	16.96
Khondab‐31	34.17	20.87	3.62	20.23	10.62	0.62	17.23
Khondab‐32	28.97	22.21	2.82	17.30	11.61	0.44	15.66
Khondab‐33	28.51	25.20	2.91	23.75	13.94	0.93	31.93
Khondab‐34	29.83	21.92	4.64	17.04	12.40	1.00	21.63
Khondab‐35	29.22	16.56	2.36	19.16	9.65	0.75	31.98
Khondab‐36	26.15	16.06	2.02	20.97	11.46	0.79	39.33
Khondab‐37	31.70	20.19	3.17	21.07	11.90	1.30	41.17
Khondab‐38	23.21	19.38	1.87	16.78	12.75	0.54	29.06
Khondab‐39	36.65	23.60	5.70	25.46	13.72	1.10	19.24
Khondab‐40	32.94	21.73	4.45	22.05	13.44	1.01	22.79
Khondab‐41	25.99	18.12	2.44	18.09	11.40	0.86	35.04
Khondab‐42	27.08	18.28	2.94	19.08	11.59	0.72	24.46
Khondab‐43	42.70	22.34	3.96	27.14	13.46	0.86	21.63
Min	20.53	15.42	1.28	12.74	7.97	0.39	10.42
Max	42.70	26.49	6.68	27.14	18.52	1.46	47.89
Mean	29.28	19.61	3.31	20.60	11.83	0.88	27.94

### 3.2 ISSR molecular marker analysis

After screening 21 ISSR primers, 8 primers were selected for PCR reactions and were used for all the genotypes. The ISSR primers used and the informativeness obtained in the studied late‐blooming almond are shown in Table 2. The primers used produced a total of 80 bands in the studied germplasm, of which 73 showed polymorphic bands.

**TABLE 2:** ISSR primers used and informativeness obtained in the studied late‐blooming almond.

Primer pairs	TB	PB	PP (%)	Rp	PIC	MI
UBC810	8	7	87.50	3.50	0.28	1.80
UBC812	7	6	85.71	3.10	0.33	2.23
UBC818	9	9	100.00	3.70	0.29	3.13
UBC834	11	10	90.91	3.50	0.18	3.80
UBC‐840	12	11	91.67	3.70	0.21	3.92
UBC868	13	12	92.31	4.10	0.23	3.62
UBC873	8	7	87.50	2.90	0.27	2.91
UBC880	12	11	91.67	3.10	0.21	3.23
Total	80	73	–	–	–	–
Mean	10	9.125	90.91	3.45	0.25	3.08

Abbreviations: MI, marker index; PB, polymorphic bands; PIC, polymorphic information content; PP, polymorphism percentage; Rp, resolving power; TB, total bands.

The MRA between the polymorphic bands and the investigated morphological traits showed that some ISSR markers have a significant correlation with these traits (Table 3). In total, 25 markers showed significant correlations with nut length, 20 markers had positive correlations, and 5 markers had negative correlations. Among them, the UBC810_1695_ marker had the highest justified *r* ^2^ and *β* (Table 3). Also, 18 markers showed significant correlations with nut width, of which 13 markers had positive correlations and 6 markers had a negative correlation with that trait. Among them, the highest values of justified *r* ^2^ and *β* were related to the UBC880_1235_ marker (Table 3). In addition, 29 markers showed significant correlations with nut weight, of which 24 markers had positive correlations and 5 markers had negative correlations. Among them, the UBC810_850_ marker had the highest justified *r* ^2^ and *β* (Table 3).

**TABLE 3:** The results of MRA between ISSR marker polymorphic bands and important fruit traits of the studied late‐blooming almond.

Trait	Number of informative markers	*r*	The highest justified *r* ^2^	The highest *β*	The marker with the highest justified *r* ^2^	The marker with the highest *β*
Nut length	25	1	0.25	0.55	UBC810_1695_	UBC810_1695_
Nut width	18	0.99	0.43	0.57	UBC880_1235_	UBC880_1235_
Nut weight	29	1	0.42	−0.71	UBC810_850_	UBC810_850_
Kernel length	19	0.99	0.44	0.95	UBC810_490_	UBC810_490_
Kernel width	10	0.95	0.32	0.74	UBC868_340_	UBC868_340_
Kernel weight	30	1	0.23	0.72	UBC868_335_	UBC868_335_
Kernel percentage	17	0.99	0.47	−0.65	UBC810_1770_	UBC810_1770_

**FIGURE 1:**
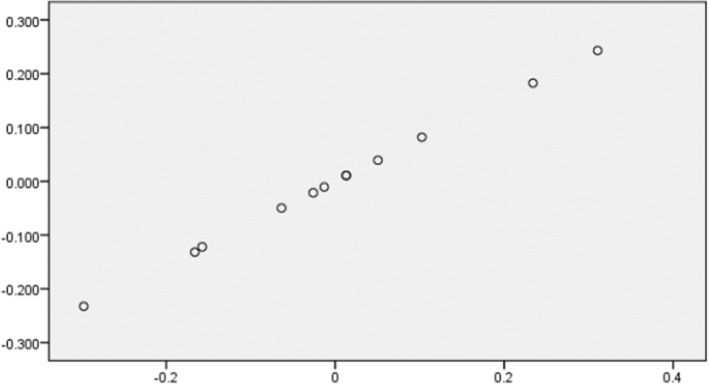
Regression plot for kernel weight with ISSR marker UBC868_335_. The *y*‐axis variable is kernel weight, and the *x*‐axis variable is residuals from regressing UBC868_335_ against the remaining independent variables.

Regarding kernel‐related traits, 19 markers showed significant correlations with kernel length, of which 15 markers had positive correlations and 4 markers had negative correlations. Among them, the highest values of justified *r* ^2^ and *β* were related to the UBC810_490_ marker (Table 3). Kernel width showed significant correlations with 10 markers, of which 6 markers had positive correlations and 5 markers had negative correlations. The highest values of justified *r* ^2^ and *β* were related to UBC868_340_ marker (Table 3). Kernel weight showed significant correlations with 30 markers, of which 24 markers had positive correlations and 6 markers had negative correlations. The highest values of justified *r* ^2^ and *β* were related to the UBC868_335_ marker. The association between kernel weight and the UBC868_335_ marker is shown in Figure 1. Also, 17 markers showed significant correlations with kernel percentage, of which 12 had positive correlations and 5 had negative correlations. The highest values of justified *r* ^2^ and *β* were related to the UBC810_1770_ marker (Table 3).

### 3.3 RAPD molecular marker analysis

After screening 25 RAPD primers, 13 primers were selected for PCR reactions and were used for all the genotypes. The RAPD primers used and the informativeness obtained in the studied late‐blooming almond are shown in Table 4. This number of primers produced a total of 181 DNA fragments, of which 151 fragments showed polymorphism among genotypes.

**TABLE 4:** RAPD primers used and informativeness obtained in the studied late‐blooming almond.

Primer	TB	PB	PP (%)	R*p*	PIC	MI
TIBMBC04	18	15	83.33	4.23	0.28	3.82
TIBMBC08	14	12	85.71	3.62	0.23	2.82
TIBMBC15	15	12	80.00	3.81	0.25	2.34
TIBMBC17	14	11	78.57	5.23	0.28	3.32
TIBMBC19	16	13	81.25	4.23	0.34	3.45
TIBMBB‐04	13	12	92.31	4.38	0.31	3.21
TIBMBB‐05	12	11	91.67	3.71	0.35	2.91
TIBMBC‐02	10	8	80.00	2.91	0.27	2.21
OPE‐13	14	11	78.57	3.43	0.29	3.90
OPN‐13	13	11	84.62	4.22	0.21	3.25
OPAB‐04	12	9	75.00	4.12	0.28	2.25
OPE‐17	14	12	85.71	3.81	0.33	2.90
OPN‐08	16	14	87.50	2.92	0.25	3.78
Total	181	151	–	–	–	–
Mean	13.92	11.62	83.40	3.89	0.28	3.09

Abbreviations: MI: marker index; PB: polymorphic bands; PIC: polymorphic information content; PP: polymorphism percentage; R*p*: resolving power; TB: total bands.

Using MRA between each of the morphological traits with 172 RAPD polymorphic bands, RAPD markers associated with each of the fruit traits were identified (Table 5). In total, 25 markers showed significant correlations with nut length, 18 markers had positive correlations, and 7 markers had negative correlations. Among them, the TIBMBC04_1230_ marker had the highest justified *r* ^2^ and the TIBMBC15_1440_ marker showed the highest *β* (Table 5). Also, 29 markers showed significant correlations with nut width, of which 22 markers had positive correlations and 7 markers had a negative correlation with that trait. Among them, the TIBMBC17_1730_ marker had the highest values of justified *r* ^2^ and *β* (Table 5). In addition, 24 markers showed significant correlations with nut weight, of which 18 markers had positive correlations and 6 markers had negative correlations. Among them, the TIBMBC17_1670_ marker had the highest justified *r* ^2^ and theTIBMBC19_1560_ marker showed the highest *β* (Table 5).

**TABLE 5:** The results of MRA between RAPD marker polymorphic bands and important fruit traits of the studied late‐blooming almond.

Trait	Number of informative markers	*r*	The highest justified *r* ^2^	The highest *β*	The marker with the highest justified *r* ^2^	The marker with the highest *β*
Nut length	25	1	0.29	0.69	TIBMBC04_1230_	TIBMBC15_1440_
Nut width	29	1	0.44	0.67	TIBMBC17_1730_	TIBMBC17_1730_
Nut weight	24	1	0.40	−0.78	TIBMBC17_1670_	TIBMBC19_1560_
Kernel length	30	1	0.38	0.85	TIBMBC15_1350_	TIBMBC19_1645_
Kernel width	26	1	0.33	0.88	TIBMBC08_1645_	TIBMBC08_1645_
Kernel weight	27	1	0.35	−0.93	TIBMBC19_1480_	TIBMBC08_1710_
Kernel percentage	29	1	0.19	−0.90	TIBMBC19_1560_	TIBMBC19_1480_

**FIGURE 2:**
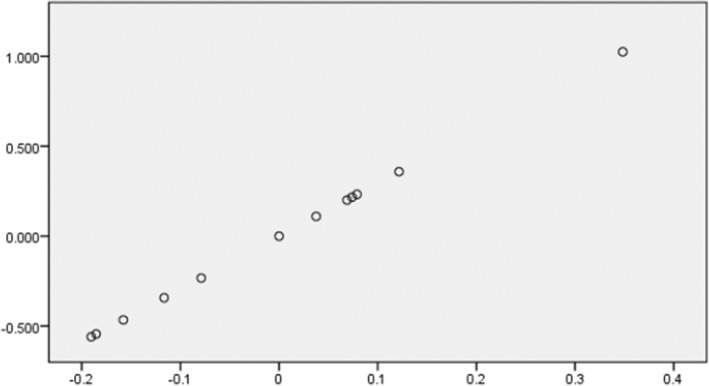
Regression plot for kernel weight with RAPD marker TIBMBC08_1710_. The *y*‐axis variable is kernel weight, and the *x*‐axis variable is residuals from regressing TIBMBC08_1710_ against the remaining independent variables.

Regarding kernel‐related traits, 30 markers showed significant correlations with kernel length, of which 24 markers had positive correlations and 6 markers had negative correlations. Among them, the TIBMBC15_1350_ marker had the highest justified *r* ^2^, and the TIBMBC19_1645_ marker showed the highest *β* (Table 5). Kernel width showed significant correlations with 26 markers, of which 19 markers had positive correlations and 7 markers had negative correlations. Among them, the TIBMBC08_1645_ marker had the highest values of justified *r* ^2^ and *β* (Table 5). Kernel weight showed significant correlations with 27 markers, of which 21 markers had positive correlations and 6 markers had negative correlations. Among them, the TIBMBC19_1480_ marker had the highest justified *r* ^2^ and the TIBMBC08_1710_ marker showed the highest *β*. The association between kernel weight and the TIBMBC08_1710_ marker is shown in Figure 2. Also, 29 markers showed significant correlations with kernel percentage, of which 19 had positive correlations and 10 had negative correlations. Among them, the TIBMBC19_1560_ marker had the highest justified *r* ^2^ and the TIBMBC19_1480_ marker showed the highest *β* (Table 5).

## 4 DISCUSSION

The results showed that the studied almond genotypes have a high diversity in terms of fruit traits and molecular aspects, and as a result of this high diversity, the relationship between molecular markers and fruit traits can be traced through MRA. Some of the bands produced by ISSR and RAPD markers showed significant correlations with the fruit traits. The location of these markers inside the genome is probably in the regions of the genome that encode the genes related to the desired fruit traits (Virk et al., 1996). Several markers were correlated with each trait, and the marker with the highest *r* ^2^ was considered the most effective marker in coding that trait (Kar et al., 2008).

Some markers were associated with more than one fruit trait, showing that the association of a marker with more than one trait can be caused by the pleiotropic effects of QTLs related to each other in different traits (Culp et al., 1979; Meredith & Bridge, 1971), but for more information from this relationship, creating a diverging population and mapping its linkage can be useful (Ebrahimi et al., 2011). The pleiotropic effect occurs when a gene can have an effect on the occurrence of several traits at the same time. Also, QTLs related to each other that control different traits can lead to the creation of a single marker that is correlated with more than one trait (Meredith & Bridge, 1971). For instance, in the present study, among the ISSR markers, UBC880_1345,_ UBC868_370_, UBC810_1440_, and UBC868_470_ showed significant correlations with nut weight and kernel weight, which indicates a positive correlation between these two traits. Also, among RAPD markers, TIBMBC15_1440_, TIBMBC08_1710_, TIBMBC19_1530_, and TIBMBC17_1800_ markers showed positive correlations with these two traits. A significant positive correlation between nut weight and kernel weight has been previously reported in almonds (Asgari & Khadivi, 2021; Heidari et al., 2022; Khadivi‐Khub & Osati, 2015).

The MRA is a suitable and quick method to find the relationship between traits and markers (Ruan et al., 2009). The markers identified in this study that have shown significant correlations with fruit traits can be used in MAS breeding programs. The obvious advantage of this analysis is that it can track quantitative trait loci (QTL). Also, it requires less time and cost (Ruan et al., 2009) and does not need to form a population for mapping (Virk et al., 1996).

The initial selection of desirable traits related to fruit and flowers requires the growth of the plants and their entry into the maturity stage. In other words, the fruit trees must go through a long juvenile stage and enter the fruiting stage so that these traits can be examined and the plants with desirable traits in terms of flowers and fruits can be found (Virk et al., 1996). However, by tracking the markers related to these traits, there is no need for the plants to enter the maturity stage. In other words, for woody plants, such as fruit trees, with a long juvenile period, it is difficult to select the superior progeny in terms of important flower and fruit traits, but by identifying the trait‐dependent markers (MAS), it is possible to identify and select the superior progeny in the early stages of their growth (Virk et al., 1996). The working method is that polymorphic DNA fragments identified as informative markers for the trait under study, such as traits related to fruit, can be separated from the gel and cloned. The identified sequence is then aligned with the existing sequences in the NCBI database and identified as candidate genes that have a high similarity to the desired informative markers. It is also possible to design SCAR primers from the obtained sequence and use them in breeding programs through trait‐dependent marker (MAS) selection (Ruan et al., 2009).

The identification of molecular markers related to the main genes controlling the desired traits has been done in recent years by creating differentiating populations such as F1 in heterozygous plants and F2, RIL, and DH in homozygous plants. Some of these markers have been used to perform breeding programs, but the unavailability of diverging populations for mapping, the lack of sufficient time, and the lack of sufficient correlation between morphological traits and molecular markers are among the most important limitations in the field of identifying markers related to morphological traits, but performing MRA lacks these limitations (Gupta et al., 2005).

## 5 CONCLUSION

It can be concluded that the informative markers identified related to fruit characteristics in almonds can be a suitable guide to identifying the genotypes with valuable fruit traits. In breeding programs, choosing quality plant materials usually takes a lot of time and cost. However, informative markers identified can be useful in selecting superior genotypes, especially when information about their genetic basis, such as a linkage map, is not available. Also, these markers can be used to select suitable parents for population generation for mapping purposes.

## AUTHOR CONTRIBUTIONS

**Ali Khadivi:** Formal analysis (lead); investigation (equal); methodology (lead); software (lead); supervision (lead); validation (lead); writing – original draft (lead); writing – review and editing (lead). **Zeinab Mashhadi:** Investigation (equal). **Akram‐Sadat Hosseini:** Investigation (equal).

## CONFLICT OF INTEREST STATEMENT

None.

## Data Availability

The data that support the findings of this study are available from the corresponding author upon reasonable request.
